# Cancer-on-a-Chip: Models for Studying Metastasis

**DOI:** 10.3390/cancers14030648

**Published:** 2022-01-27

**Authors:** Xiaojun Zhang, Mazharul Karim, Md Mahedi Hasan, Jacob Hooper, Riajul Wahab, Sourav Roy, Taslim A. Al-Hilal

**Affiliations:** 1 Department of Pharmaceutical Sciences, School of Pharmacy, University of Texas at El Paso, El Paso, TX 79968, USA; xzhang5@miners.utep.edu (X.Z.); mmkarim@utep.edu (M.K.); mhasan11@miners.utep.edu (M.M.H.); riajulwahab@yahoo.com (R.W.); 2 Department of Biological Sciences, College of Science, University of Texas at El Paso, El Paso, TX 79968, USA; jhooper2@miners.utep.edu (J.H.); sroy1@utep.edu (S.R.); 3 Department of Environmental Science & Engineering, College of Science, University of Texas at El Paso, El Paso, TX 79968, USA; 4 Border Biomedical Research Center, University of Texas at El Paso, El Paso, TX 79968, USA

**Keywords:** cancer-on-a-chip, microfluidic chip, metastasis-on-a-chip, cancer cell migration, metastatic microenvironment

## Abstract

**Simple Summary:**

Microfluidic-based cancer-on-a-chip models are powerful tools to study the tumor microenvironment (TME). Two-dimensional cell culture cannot recapitulate TME. In vivo animal models can better represent the TME, but their physiology is vastly different from that of humans. Although three-dimensional tumor models can bridge the gap between in vitro and in vivo examination, they are still unable to test many crucial cues from the TME, such as mechanical cues, cell–cell, and cell–extracellular interactions. Cancer-on-a-chip platforms enable studying the metastatic process in a step-wise manner with precise control. We present an overview of the recent advances in cancer-on-a-chip models on metastasis including models that mimic mechanical cues. This review article will provide knowledge of the latest progress made on cancer-on-a-chip models.

**Abstract:**

The microfluidic-based cancer-on-a-chip models work as a powerful tool to study the tumor microenvironment and its role in metastasis. The models recapitulate and systematically simplify the in vitro tumor microenvironment. This enables the study of a metastatic process in unprecedented detail. This review examines the development of cancer-on-a-chip microfluidic platforms at the invasion/intravasation, extravasation, and angiogenesis steps over the last three years. The on-chip modeling of mechanical cues involved in the metastasis cascade are also discussed. Finally, the popular design of microfluidic chip models for each step are discussed along with the challenges and perspectives of cancer-on-a-chip models.

## 1. Introduction

The primary cause of cancer death results from metastasis, which causes up to 90% of cancer-related mortality [1]. Metastasis—a multistep process—is the migration and spread of cancer cells from the initial tumor to distant organs as well as their uncontrolled growth [2,3]. In the metastasis process, both collaborative and antagonistic interactions between host microenvironmental factors and tumor cells take place [4]. Two-dimensional (2D) cell culture and animal models are traditional cancer investigation models, but these cannot recapitulate the tumor microenvironment (TME) that is relevant to human physiology [5,6]. Although three-dimensional (3D) tumor models including tumor spheroids and hydrogel systems bridge some of the gaps between in vitro and in vivo findings, they are still unable to test many critical cues presented in the 3D TME, such as physical and mechanical forces or compartmentalized architecture of primary or secondary tumors that house both tumor and non-tumor cells.

The recently developed cancer-on-a-chip models, containing small chambers, are evolving as powerful tools to investigate the TME and its contribution in metastasis [7]. These approaches capitalize on specialized multichannel system to simulate the mechanics, activities and physiological responses [5]. The biochemistry and geometry of tumors can also be engineered on these novel “on-chip” platforms. The fluidic transport and response to soluble factors can be mimicked through microfluidic channel resembling vasculature networks [8]. The capability of simulating gradient factors (such as fluidic flow, oxygen, drug), tissue mechanics, tissue engineering, and local environmental compositions enable these “on-chip” platforms as powerful tools for studying the complicated interactions that are involved in the metastatic process [9]. Thus, with an accuracy level that cannot be reached out by conventional models, the functional “cancer-on-a-chip” models can be designed for studying TME and cell–cell interactions during metastasis.

In this review, key representative microfluidic platforms have been collated by cues and cancer types to address the most important steps of the metastatic cascade. The main contribution of this review is the collation of key representative platforms, sorted by cues and cancer types. The respective chip designs for each step of cancer progression are highlighted. Notably, we discussed the tumor microenvironmental biophysical and mechanical factors that are hard to recapitulate in conventional models but can be integrated into the microfluidic system. Also, the challenges and future perspectives are discussed in a technological and pathophysiological perspective. The metastatic processes involving TME are briefly introduced in Section 2. Then, the microfluidic-based tumor invasion/intravasation, extravasation, and angiogenesis on-chip models are overviewed in Section 3. We also discuss how the “on-chip” models are designed to simulate the mechanical forces, and to study the role of cellular and non-cellular components during cancer cell dissemination in Section 4. Finally, the challenges and future perspectives are discussed in Section 5. This review will help us to understand the need for the design of a suitable, pathophysiology-driven cancer-on-a-chip model to gather further knowledge on a tumor evolution process, the complex interplay between cancer biological and biophysical signaling, collaborative mechanisms between cancerous and non-cancerous cells, and screening drugs for personalized therapy. We envision that combining the cancer-chip models with the state-of-the-art techniques such as additive manufacturing (3D bioprinting), computational fluid dynamics, artificial intelligence, single cell RNA sequencing, and high-throughput omics would make the models very effective in fundamental studies of cancer research.

## 2. Overview of the Metastasis Process

**Figure 1:**
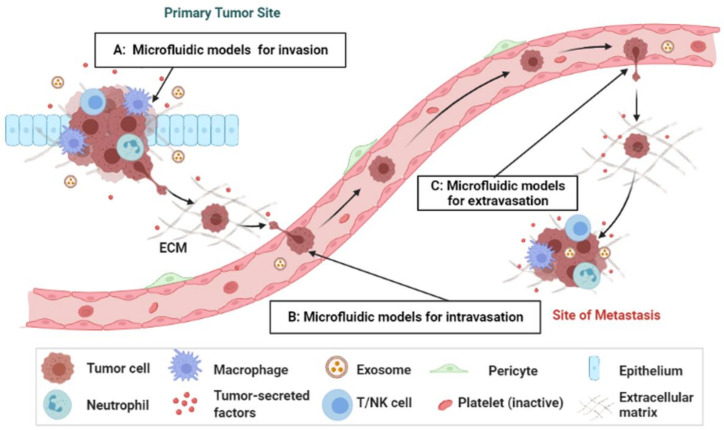
Microfluidic models involving metastatic steps described in this paper. (**A**,**B**) Invasion/intravasation process and (**C**) extravasation process.

Metastasis involves three primary processes: invasion/intravasation, extravasation, and angiogenesis (Figure 1). Tumor cells invade through the extracellular matrix (ECM) or vascular endothelium into the circulation, then extravasate and colonize into other organs, and form micrometastases [10,11,12]. Because of the vascular system, the endothelial wall represents a natural barrier to the migrating tumor cells that need to be breached during the intra-/extravasation process [13]. Intravasation is a process where tumor cells travel beyond the endothelial basement membrane of blood vessels to enter the circulation. Extravasation is a process where tumor cells travel across the vessel wall to leave the circulation and enter into a metastatic site/organ. It is the last and rate-limiting step before secondary tumor formation, which plays a vital role in cancer development and metastasis.

In cancer metastasis, an early and key event is the shedding of tumor cells that eventually intravasate into the blood circulation. However, only less than 0.01% of circulating tumor cells (CTCs) develop into metastases [14,15]. Isolation and enumeration of CTCs, by microfluidic technologies, have been promising for early diagnosis, and predicting prognosis and patient-centric treatment of cancers [2,10,16]. The clinical identification of metastatic breast, prostate, and colorectal cancers using CTCs has received United States Food and Drug Administration (FDA) approval [15]. Moreover, CTC clusters (aggregated CTCs) are more invasive and metastatic [17]. However, the invasion, intra/extravasation, and re-growth of CTCs into secondary organs involve complex interactions between tumor cells and TME that cannot be deciphered by conventional methods but with the microfluidic-based cancer-on-a-chip models.

## 3. Microengineered Metastatic Models

**Figure 2:**
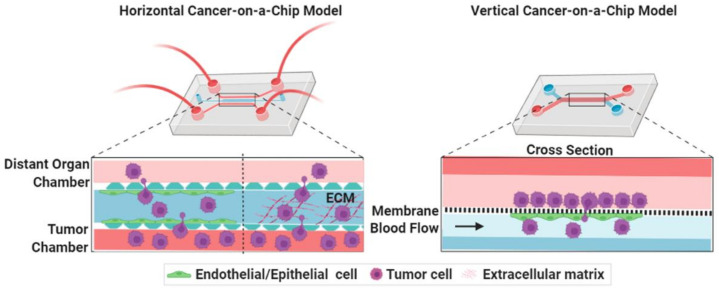
Simple representative microfluidic-based cancer-on-a-chip models show the movement of cancer cells from its site of origin to the distant organs. Microfluidic-based cancer-chips models are grossly classified into two types: horizontal and vertical chips. In horizontal cancer chip models (such as microvascular network (MVN) chip), the chambers are walled off by micron-sized pillars, which creates separate compartments for growing different cell types in their own zone without mixing with each other during the initial seeding but allow cellular interactions via paracrine, juxtracrine, or mechanical fashion. In vertical chips, the channels are separated by a membrane that may represent both cancer intravasation and extravasation process. In some cases (such as ovarian TME organ-on-a-chip (OTME)), a vertical layer can be integrated with the horizontal chips to mimic a more complex tumor pathophysiology.

The TME of a metastasis process can be classified into three groups: the primary TME, circulation microenvironment, and the secondary TME [18]. To enter the circulation, cancer cells must disintegrate the ECM and subsequently intravasate across the endothelium layer. At the distant organs, survival CTCs get physically trapped into the small vessels, disintegrate the ECM, and proliferate to form a secondary tumor [1]. The secondary TME is markedly different than its primary site, where metastatic cells should adapt the microenvironment of secondary tumor site [11]. TME also includes stromal components, such as tumor vasculatures, mesothelial cells, immune cells, fibroblasts, ECM and lymphatics (including endothelial cells and pericytes cells) [5]. Thus, metastasis models should contain both tumor and non-tumor cells, external/mechanical cues, and be able to measure their functions (Table 1). Simple representative microfluidic models are classified into two types: horizontal and vertical chips for study cancer models (Figure 2). Both chips are designed to maintain the communication between tumor with non-tumor cells by paracrine, juxtracrine or mechanical signaling pathways. The micron-sized channels inside the microfluidic devices create a more biologically relevant microenvironment for tumor cell’s growth than the conventional systems and enable cell–cell or cell–matrix communications with continuous bathing in biological fluids in a tumor tissue-like fashion. The channels can be incorporated with cellular or mechanical cues, such as fluid-induced stress, ECM gradients, and allow monitoring of cancer invasion/intravasation and extravasation processes, as well as the screening of anti-cancer drugs.

### 3.1. Cancer-on-a-Chip Models for Studying Cancer Cell Invasion/Intravasation

#### 3.1.1. Influence of Inflammatory Cells

Inflammatory cells are recruited from the circulation by cancer and stromal cells. An intricate network of immune cells, such as natural killer (NK) cells, myeloid-derived suppressor cells, regulatory T cells, and tumor- associated macrophages (TAM) in the TME contribute to tumor progression. TAM has the ability to enhance proliferation of tumor cells, promote metastasis and angiogenesis, and regulate T-cell functions that are linked with poor prognosis in cancer patients [43]. M1 macrophages have pro-inflammatory and anti-tumorigenic properties. However, with the tumor progression, M1 macrophages polarize towards M2 phenotype with immunosuppressive and pro-tumorigenic activities. The compartmentalized features of microfluidic chips endow them to study the communication between tumor cells and macrophages, and the transformation of M1 to M2 phenotypes inside the TME.

Tumor cells and macrophages synergistically affect each other, as shown by a juxtaposed dual-layer, cell-loaded biomimetic microfluidic system that produced a tumor-macrophage bidirectional crosstalk [19]. U937 cells, a type of monocyte, changed their phenotype to TAMs after culturing with IL-4. Both the invasion number and the migrating capability of invasive breast cancer cells were promoted by TAM into the adjacent channel. The phenotype of TAMs was maintained by breast cancer cells whereas breast cancer cells promoted the differentiation of U937 cells into TAM, suggesting a bidirectional crosstalk between them that was mimicked into the microfluidic system. Another study also showed a crosstalk between lung carcinoma cells (A549 and NCI-H1975) and macrophages by the action of αB-crystallin (CRYAB), a small molecular heat shock protein with a cytoprotective role [20]. An integrated microfluidic-based device was developed with one upper (Chamber A) and one lower (Chamber B) polydimethylsiloxane (PDMS) layer sandwiching a transwell membrane that segmented into two chambers, where cancer cells and macrophages were grown into chamber A and B, respectively. The bottom chamber (Chamber B) was also connected to another cell culture Chamber C containing two channels that were separated by micropillars and allow the monitoring of cancer cell migration and invasion from one channel to another one by the influence of macrophage-secreted factors. In this device, lung cancer cells (Chamber A) upregulated CRYAB expression by M2 macrophages (Chamber B), which in turn triggered the ERK1/2/Fra-1/slug signaling pathway to promote epithelial-to-mesenchymal transition (EMT) and metastasis of cancer cells in the chamber C.

Another microfluidic-based model revealed how the crosstalk between activated macrophages and P-selectin expressing mesothelial cells increased the adhesion of ovarian cancers cells [21]. A micro-culture device was designed with a PDMS ring that was attached with a coverslip. Within the ring, one or additional cell types can be grown while on the coverslip, another cell type can also be grown. Co-culture between the populations was initiated by overturning the coverslip up the ring. This device was utilized by examining the interactions between macrophages, tumor cells, and mesothelial cells. Macrophages secreted macrophage inflammatory protein-1β (MIP-1β) that activated CCR5/PI3K signaling in mesothelial cells and induced P-selectin expression on the cell surface. Through CD24, a surface receptor, tumor cells adhered to P-selectin expressing mesothelial cells and increased the adhesion in both static and shear conditions.

Solid TME produces many environmental stress factors, such as acidic pH, nutrient depletion, hypoxia, or accumulated waste product that blunt the action of immune response and suppress NK and T cells’ antitumor activity. To understand the effects of tumor environmental tension on NK cell functions, a cancer-on-a-chip model was designed [22]. The on-chip model includes a central microchannel where breast cancer cells were embedded in a 3D matrix with/without NK cells and connected with a lumen that was lined with endothelial cells. The existence of the lumen at the end of the microchamber led to an uneven distribution (proximal to distal) of nutrient, pH, proliferation and necrosis, and mimic the gradients across solid tumors. A culture of NK cells in the device, with environmental gradients, progressively eroded their cytotoxic ability and caused exhausted NK cells. Despite removing the environmental stress from the device, the process of NK cell exhaustion was irreversible as shown by multiple molecular and functional alterations. This work highlights the importance of on-chip cancer models in the need to improve immunotherapy by incorporating tumor-induced environmental stress factors that cannot be recapitulated by conventional 2D/3D models.

To understand the lymphatic metastasis, a three-channel microfluidic system was produced for in situ monitoring of tumor cell invasion and colonization by mimicking the lymph-tissue-blood vessel (LTB) structure [23]. Collagen-based hydrogel was filled in the middle channel to replicate in vivo tissues and human lymphatic endothelial cells (HLECs) and human umbilical vascular endothelial cells (HUVECs) were grown on the two side channels to form LTB layers. The consequences of inflammatory cytokine, IL-6, on the metastasis of breast cancer process were monitored using this LTB chip. IL-6 treatment induced EMT in the breast cancer cells. Compared to the control, IL-6-treated breast cancer cells presented with higher transvascular invasiveness, successfully immobilized and colonized onto the HLEC channel of LTB chips. Vascular endothelial growth factor (VEGF)—secreted by HLECs upon IL-6 stimulation—caused the HUVECs to grow in the direction of the cancer cell clusters that were located close to the lymphatic channel.

These studies show that microfluidic platforms are helpful to mimic and understand the cancer metastasis process and the mutual interactions between tumor cells and immune cells. Inclusion of immune of cells in the cancer-on-a-chip models might help to better understand the process of immunosuppression and thus, the development of new immunotherapies by bridging the gap between in vitro and animal models.

#### 3.1.2. Influence of Cancer-Associated Fibroblasts (CAFs)

Being one of the most abundant non-cancer cell types within the TME, CAFs have unique roles in tumor metastasis and invasion process. The crosstalk between fibroblasts and breast cancer cells (MDA-MB-231) were shown using a 3D microfluidic system in the presence of fibronectin [25]. A LumeNEXT microfluidic model was used to recreate the TME that allows easy incorporation of lumen structure with different cells and the study of complex system invasion process. The main focus was to study the impact of fibronectin on the cancer cell invasion stage in the presence of human mammary fibroblasts (HMFs) or CAFs. The lumen structure contained the MDA-MB-231 cells and the surrounding space acted as the stroma where cancer cells could invade. During co-culture of MDA-MB-231 and fibroblast cells, significant level of metalloproteinases (MMPs) secretion was noted when cancer cells started to invade the fibroblasts layer. Interestingly, the inhibition of MMP decreased the movement of MDA-MB-231 cells in all combinations except when HMFs were cultured with fibronectin, indicating an important role of fibronectin in altering HMFs to CAFs. The microfluidic-based model highlights that both MMP inhibition and the regulation of fibronectin are essential to stop the invasion of MDA-MB-231 cells. Thus, understanding of fibronectin effect during metastasis process could lead to a better clinical trial design of MMP inhibitors [25].

The role of CAF-differentiation into the TME and their role in tumor invasion were also modelled. Differentiation of endothelial cells into CAFs can be stimulated by cancer cell-derived exomes that transfer molecular signals to surrounding cells; a 3D microfluidic model was fabricated for the real-time monitoring of this process. Endothelial cell-derived CAFs, induced by exosomes, were isolated and grown into the device in the presence of different cancer cell lines, such as human breast cancer cells, human squamous cells, and mouse melanoma [28]. The invasive area of cancer cells into the ECM in the existence of endothelial cell-derived CAFs was higher than the non-endothelial cell-derived CAFs. Invasive melanoma, breast, and squamous cancer cells were associated with increased AKT1, IL-6, and EGFR signaling, respectively.

Three-dimensional cellular models can mimic the in vivo condition better than 2D models. However, the conventional 3D models cannot truly mimic the complex TME, thus are inadequate for understanding the role of fibroblast cells in metastasis process [27]. A 3D printed brick like cell patterning microfluidic platform was developed for studying the effect of tumor cells on fibroblasts through co-culturing process [26]. The study showed that almost all fibroblast cells converted to an intermediate state between normal and CAFs, which was associated with hyperactivity of ribosome biogenesis. At the end, these intermediate fibroblasts were converted to CAFs. Deep data mining combined with in vivo results indicated that both the tumor cells and fibroblasts crucially impacted each other. While the HT1080 cells converted the fibroblasts to CAFs, the surrounding fibroblasts controlled the spread of fibrosarcoma cells at the initial stage of metastasis [26]. Another microwell array-based microfluidic platform was designed to elucidate how tumor spheroids invade in the presence of fibroblasts. Each device was capable of pairing with 240 tumor-stroma, allowing for a large data set to analysis the invasion process. Tumor spheroids could envelop fibroblast spheroids completely. To understand the metastatic ability of different tumor cells, both colon and breast cancer cell spheroids were co-cultured with fibroblast cells. Colon cancer cells invaded at a much shorter time than breast cancer cells in the existence of fibroblasts. In another study with a 3D co-culture organotypic invasion model, it was revealed that CAFs enhanced invasion through inducing gene expression of glycoprotein non-metastatic B [24]. The system resembled the architecture of the early TME and represented side-by-side bidirectional crosstalk between the tumor and stroma regions. The platforms allowed imaging-based quantifications and incorporated RNA-seq for molecular mechanism exploration.

These studies showed that cancer-on-a-chip can be used as a powerful tool to understand the crosstalk between cancer and non-cancerous cells. The recreation of complex TME allowed to identify different factors that modulate the progression of the metastasis process that is impossible to study using conventional cell culture systems.

#### 3.1.3. Use of Endothelium-Based Models

The development of microfluidic devices that closely resemble the blood vessels’ architecture (round channel-like structure with a lumen) results from the importance of tumor vessels during the intravasation process [13]. Fabrication of an endothelium-based vascularized channel within the microfluidic-based cancer models is considered a signature way to mimic the cancer intravasation process, since cancer cells invade the endothelial basement membrane before they enter the circulation. However, different cancers invade the endothelial layers differently. Using a vascularized microfluidic chip, it was shown that MDA-MB-231 breast cancer cells invaded paracellularly by disrupting the endothelial junction, whereas HepG2 liver cancer cells invaded through the transcellular process [29]. Using another in vitro model of an invasive ductal carcinoma (IDC)-on-a-chip model that was developed using viscous fingering method, invasive characteristics of different human breast cancer cell lines were identified [30]. Breast cancers have been classified into different subtypes by their different gene expression profiles, which include luminal A, luminal B, hormone epidermal growth factor receptor 2 (HER-2) enriched, basal-like, and triple-negative breast cancer (TNBC) type. TNBC lacks the expression of HER-2, estrogen receptor, and progesterone receptor. MCF-7 is the luminal A subtype breast cancer cell line expressing both progesterone and estrogen receptors. In the IDC-on-a-chip model, the MCF-7 cell line was non-tumorigenic and non-invasive unless supplemented with estrogen, whereas TNBCs (SUM-159PT and MDA-MB-231) invaded into the adjacent matrix, suggesting that different subtypes of breast cancer cells have a different potential for invasion.

Recently a novel ovarian TME organ-on-a-chip (OTME-chip) has been engineered which incorporates collagen-based ECM microenvironment in addition to the tumor interfacing platelet-perfused vascular endothelial channel [31]. This novel design facilitates the study of dynamics of cancer cell invasion following the biological and biophysical effects of platelet extravasation through the endothelial channel into the TME. The advantage of the OTME-chip over the conventional in vitro and in vivo models was demonstrated by studying the complete biomimicry effects of cancer cell-platelet interaction in ovarian cancer metastasis process. In addition, the hydrogel compartment attached to the cancer cell-vessel interface in the chip allows the perfusion of platelets in the vessel, platelet extravasation through endothelium, and the invasion of ovarian cancer cells. The design of the OTME-chip permits the time-lapse isolation of cancer cells based on the degree of platelet perfusion that allows to study the platelet-mediated alterations in cancer cell cycle and proliferation. The results showed that platelets promote ovarian cancer invasion via the interactions between glycoprotein Ⅵ and tumor gelactin-3 under flow.

These studies have shown how immune cells, CAFs and endothelial cells are involved in tumor invasion/intravasation process that can be mimicked into the microfluidic platform. However, different types of cancer cells interact differently with TME-producing cells that are involved in the intravasation process. Thus, designing complex heterotypic microfluidic chips that can incorporate various physiological factors would greatly impact our understanding of the cancer metastasis process.

### 3.2. Cancer-on-a-Chip Models for Studying Cancer Extravasation

Cancer cells need to undergo extravasation, a process by which cancer cells move to secondary tissues or organs through blood vessels to form metastases. The endothelial barrier that cancer cells need to extravasate to reach the metastatic site is a major regulator of metastasis. Cancer cells adhere to blood vessels, transmigrate through the endothelium, and finally escape the circulation to invade into the secondary site. Hence, many microfluidic based models are designed to investigate the role of surface adhesion molecules and endothelium permeability in cancer extravasation. A microfluidic platform with microvascular network (MVN) was developed as a model for studying the extravasation of tumor cells. HUVECs were seeded in fibrin gels and cultured alongside or together with normal human lung fibroblasts (HLFs) to generate MVNs in the central channel [32,33,34,35,44]; signaling between the two cell lines helps to maintain stable networks. After pefusable microvasculature formed, cancer cell lines were introduced into the vascular networks. Exploiting the vascularized microfluidic MVN chips, the role of the Cdk5/Tln1/FAK^S732^ axis in each step of cancer extravasation was studied: vascular adhesion, trans-endothelial migration, and early invasion [32]. Cyclin-dependent kinase (Cdk5) silencing primarily affects the vascular adhesion of cancer cells. Talin 1 (Tln1) and Focal Adhesion Kinase (FAK) orchestrate the actin polymerization that leads to invadopodia formation and 3D-matrix invasion. FAK^S732^ phosphorylation was identified as an essential event driving cancer cell trans-endothelial migration. Mimicking the findings in a microfluidic-based MVNs model, Tln1 and FAK inhibition significantly reduced in vivo colonization of breast cancer cells into the lungs. These findings highlight the importance of using microfluidic-based cancer extravasation models and find a new role of phosphorylation sites of FAK—such as serine 732—in the late events of a metastasis cascade.

A similar chip was used to understand the role of endothelial glycocalyx in the extravasation process. The endothelial glycocalyx is a brush-like structure forming the inner-most lining of human microvasculature that creates a repulsive barrier between the solid tissue in the perivascular space and the liquid tissue within the vessels. Glycocalyx shedding precedes cancer cell extravasation. With confocal imaging of lectin-stained MVN-chips, the glycocalyx defects during extravasation of breast cancer cells (MDA-MB-231) was visualized from perfusable endothelial lumens [33]. A function of glycocalyx was proposed where tumor cells collaborate with the endothelial glycocalyx to form an adhesive vascular niche [34]. Furthermore, hyaluronic acid that is shed by tumor cells in the MVN-chip model assists the adhesion of cancer cells to the endothelium via interaction with glycoprotein CD44. Trans-endothelium migration and invasion of cancer cells occur into the MVN stroma through binding of the isoform CD44 to the subendothelial ECM components. Significant reduction in the extravasation of tumor cells was achieved by targeting the hyaluronic acid-CD44 glycocalyx complex. Thus, microfluidic-based cancer MVN models have evolved as an important tool to study how tumor cells use the vascular glycocalyx to promote cancer progression.

Using the MVN-chip model, the role of inflamed neutrophils during hematogenous dissemination was also studied: tumor cells and LPS-stimulated neutrophils (PMNs) aggregated under flow, and arrested into the vascular channel by mechanical trapping and interaction with endothelial ICAM-1 [4]. After being arrested, PMNs were further sequestered by self-secreted IL-8 and tumor-derived CXCL-1 to form tumor cell-PMN clusters. The sequestration of PMNs at the vicinity of tumor cell clusters resulted in the spatial localization of IL-8, which disrupts endothelial barrier and enhances the extravasation of tumor cells. The impact of a subunit of hypoxia-inducible factors (HIF-1α) on cancer progression was also investigated using MVN-chip model [35]. Hypoxia increased HIF-1α level, and changed the morphology, viability, and metastatic potential of cancer cells. Compared to normoxia (21% O_2_), the rate of extravasation increased as well. Meanwhile, the extravasation rates of all tested cell lines were markedly decreased through siRNA knockdown of HIF-1α in hypoxic tumors. The above discussion highlights that multiplexed microfluidic based cancer extravasation models can be powerful tools to study the transport of circulating cells and real-time monitoring of heterotypic cell–cell interactions.

### 3.3. Cancer-on-a-Chip Models for Studying Tumor-Associated Angiogenesis

**Figure 3:**
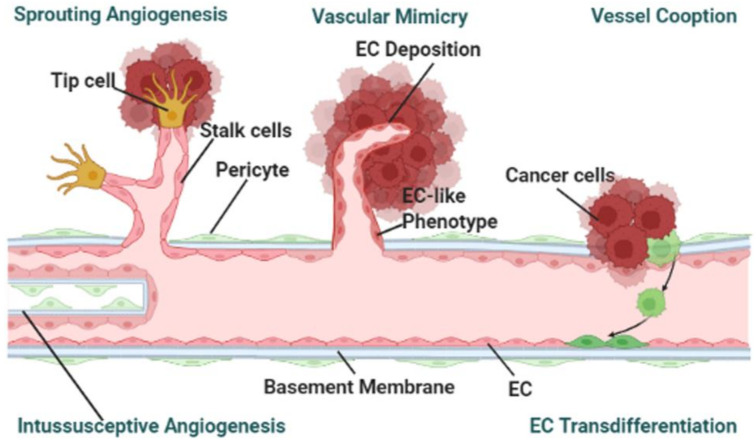
Tumor endothelial cells vascularize in different mechanisms: During sprouting angiogenesis, endothelial cells respond to proangiogenic factors to form a dynamic of stalk and migrating tip cells that control and guide the sprouts. Intussusceptive angiogenesis forms hollow transcapillary pillars and splits into 2 parallel vessels. In vessel co-option, cancer cells collaborate with adjacent normal tissue vasculatures and thus, incorporate the vessels into the tumor. In some cancers, a non-endothelial blood irrigation system is developed where cancer cells eventually transdifferentiate into endothelial cells, a process known as vascular mimicry. Cancer-on-chip models are heavily concentrated on articulating the process of sprouting angiogenesis in tumors.

Angiogenesis is a procedure by which the growth of new blood vessels are formed from the pre-existing blood vessels [38]. Designing cancer-on-a-chip models with perfused microvessels that can represent endothelial cell lining, angiogenesis or vasculogenesis is still a great challenge [45,46]. In response to the cellular microenvironment and biochemical gradients, pre-existing vessels grow new vessels. Responding to pro-angiogenic stimuli, endothelial cells differentiate into three characteristic phenotypes: endothelial tip, stalk, and phalanx cells (Figure 3). Endothelial tip cells invade the matrix while stalk cells form the lumen. To form a robust and directional vasculature with angiogenic sprouts and a clear lumen, a three-lane microfluidic titer plate was designed [36]. The angiogenic sprouts formed by endothelial cells were initially leaky and then became leak-free once they fully crossed the collagen lane. Since the perfusion in the device was induced by a rocker platform, the flow was bidirectional contrary to physiological flow, which is continuous and unidirectional. The assay was also limited to performing time-lapse imaging only at discrete time points. Although the precise contribution of flow in this assay system is hard to determine, the flow influences the capillary remodeling and maturation.

It is important to investigate the mechanism of angiogenesis at a very early stage to prevent capillary formation. A cervical tumor spheroid-based microfluidic device consisting of three compartments was designed [37]. The tumor progression at the very early stage, when tumors are too small to be identified by imaging, was studied by seeding HeLa cell-spheroid on the chip. The proliferation and migration of HeLa cells in MVN chips were accelerated by endotheliocytes and fibroblasts. The smaller tumors showed more aggressive phenotype. For the first time, the EMT and its role in phenotyping the vascular mimicry process during tumor progression was modelled in the chip. Vasculogenic mimicry is the formation of vessel-like structures without endothelial cells that represents an alternative pathway for tumor cells to gain enough blood supply and nutrients. The presence of vasculogenic imitation in cervical cancer on-a-chip displayed as HeLa cells stretched and interconnected with each other to form a tube-like structure.

VEGF plays an important role in vascular sprouting and microvascular network formation. In the cancer-angiogenesis chips, interstitial flow increased vascular sprout formation, network extension, and branching [38]. The migration of tip cells—which led to network extension—is supported by interstitial flow, while the proliferation of stalk cells is promoted by VEGF. The balance between the interstitial flow and VEGF concentration played a critical role in the regulation of 3D MVN formation in vivo and on-chip models. Contrary to rectangular lumen channel chips, a round lumen microfluidic chip was fabricated by pre-casting a needle before polymerization of injected ECM hydrogel that was supplemented with tumor spheroids. A vascular channel was created following the post-removal of the needle and culturing of HUVECs that grew into an intact perfused layer [41]. Culturing of MDA-MB-231/HUVECs spheroids in a fibroblast-laden, fibrin-based ECM, within the microfluidic device preserved the integrity of the endothelial cell channels while simultaneously promoting the tumor cell’s migration and active patterns of angiogenesis. Several proangiogenic growth factors promoted the new vascular sprouting and preserved the existing blood vessels’ structural integrity. The study established that the combination of fibroblast-secreted growth factors, such as HB-EGF, VEGF, and PIGF created a pro-angiogenic cell culture microenvironment.

Compared to normal fibroblasts, CAFs drive angiogenesis. One complex microfluidic-based platform was developed containing three independently separated microtissue chambers that included one central chamber adjacent to other chambers and two lateral media lines [40]. The separate media lines, for every tissue chamber, allowed directional interstitial flow between microtissues. The central chambers were filled with normal HLFs and endothelial cells, while side chambers were filled with stromal cells (normal breast fibroblasts or CAFs) or cell-free fibrin gels (controls). Two regimes were used: interstitial flow domination as top to bottom and convection domination as outward flow. The two sets of flow parameters efficiently controlled crosstalk between microtissues. By controlling the effects of interstitial and outward flow on vessel chamber in the chips, the mechanical activity of CAFs was also controlled that contributed to increased angiogenesis.

With a pre-casting 22G needle method, a 3D vascularized inflammatory breast cancer (IBC)-on-a-chip was developed [42]. Compared to ten years median survival of non-IBC cases, IBC cases have a median survival of 4 years. The platform was specific to IBC tumor–ECM interactions and tumor-vasculature. The platform was cultured with non-IBC cells (MDA-MB-231) and IBC cells (HER2+ or SUM149) that contained collagen type I ECM with endothelialized blood vessels. An acellular platform was served as control. Compared to control, chips containing SUM149, and MDA-MB-231 cells decreased the vessel lumen endothelial coverage, while HER2+ and SUM149 increased collagen ECM porosity and expressed higher levels of VEGF. Furthermore, vessels sprouting was observed which led to viable vessels within the lumen of IBC platform.

In another model, in which endothelial cells were cultured in a hydroxyapatite (HA)-rich TME composite, the number and lengths of angiogenetic sprouts were short [39]. HA, a constituent of bone ECM, has unique mechanical properties, which affects the formation of angiogenetic sprouts in the bone microenvironment. HA nanoparticles were kept in the fibrin ECM of the microfluidic chip to study the HA-TME interactions. The mixture of HA/fibrin composite, HLFs, and SW620 or MKN74—two highly metastatic tumor cells—were patterned into one side channel. HUVECs were cultured at the far side boundary of the central channel that was separated by one media channel by tilting the microfluidic chip at 90 degrees. Tumor-fibroblast spheroids were also patterned into the central channel using HA/fibrin composites and endothelial cells were attached to the wall of the middle channel. The number of vessel sprouts decreased during angiogenesis of tumor-stromal cell spheroids in the HA/fibrin composite. With an increase of HA concentration, a biphasic response was exhibited on the sprout length.

The conventional methods so far developed to study in vitro angiogenesis assays are mostly based on growing endothelial cells on 2D cell culture dishes, lacking the adequate cell–cell and cell–matrix interaction resulting in poor information applicable to actual physiological conditions. The in vitro assay systems, based on microfluidic chip models, reflect the complete cascades involved in angiogenesis and would be more effective to screen different pro- and anti-angiogenesis factors influencing the entire process.

## 4. Microfluidic Modeling: A Focus on Mechanical Factors

### 4.1. Mechanical Factors in Cancer Cell Migration

In the microfluidic chips, the fabricated microchannels can be used to precisely control the interstitial fluidic flow or wall shear stress, pressure, etc. while networks can be made to closely resemble small capillaries in size and branching by integrating with various microelements [19,47]. The ECM is the non-cellular element of tissues and organs that provide mechanical and chemical support to the cells [48]. With the support of ECM components, the cells are cultured in 3D morphology to maintain polarity. Like other cell types, from their microenvironment, tumor cells recognize physical inputs that mechanically alter DNA transcription, cellular behavior, and function, a procedure known as mechanotransduction [49]. The mechanical features of TME have been characterized by altered stiffness, shear stress from fluidic flow, porosity, deformation, the architecture of the ECM (narrow constrictions, interstitial pores), and so on [7,50]. From stiffness changes to the supply of nutrients through blood flow, mechanical cues play a variety of roles in cancers [51]. Thus, the importance of anomalous mechanical properties of tumor tissue has become apparent. Excessive matrix remodeling, driven by TME, leads to the changes in ECM physical properties, which have been linked to cell migration, malignant transformation, and EMT activation [52]. In response to these mechanical cues, the protein expression, signaling pathways and the differentiation of cancer cells are known to change [53]. The microfluidic platform is an attractive tool to mimic and control multiple features of mechanical cues that cannot be translated by the current in vivo or in vitro methods [54,55]. The impact of mechanical cues on cancer cell migration and how they are translated by the microfluidic chips (Table 2) are discussed in the following section.

#### 4.1.1. Simulating Stiffness in the Microfluidic-Based On-Chip Models

Due to fibrosis in the advanced stages of a tumor, the collagen density and stiffness are increased in the tumor stroma. Reciprocally, exogenous tissue stiffening can facilitate tumorigenesis [1]. The high tumors stiffness induces the mechanical activation of biochemical pathways that enhance the cell cycle, cell motility, and EMT pathways. During metastasis, cancer cells pass through the ECM pores as small as 1 µm at the primary and secondary tumor sites as well as the capillaries or small lymphatic vessels (5–10 µm). The different confinement, stiffness and mechanical cues of primary and secondary tumors are known to affect cancer cell migration.

To understand the effect of substrate stiffness on the primary migration of cancer cells, a microfluidic-based on-chip model containing cancer cells, endothelial cells, and physio-mechanical properties of the ECM was employed [56]. The microfluidic device contained three independently addressable parallel channels while the biological matrix (collagen solution) was injected into the central channel. In the migration model, cancer cells (invasive MDA-MB-231 and non-invasive MCF7) were seeded into the side channel whereas in the extravasation model, both endothelial (HUVECs) and cancer cells were loaded into the side channel under the condition of different stiffness substrates. Different stiffnesses of PDMS substrates were utilized by mixing the silicone elastomer with the curing agent at three different ratios. The results showed that cancer cells markedly increase their extravasation capacity in response to the substrate stiffening which could be correlated with the expression level of MMP-9. However, the shortfall of this model is the lack of a long-term culture condition of cancer cells that limits the possibility of examining the colonization of cancer cells.

Benign cancer cells are more rigid than their metastatic ones. The cellular rigidity, as determined by the stiffness, proportionally decreases with cancer progression, whereas the nanomechanical stiffness inversely correlates with the cancer cells migration potential. Using a linearly tapered microflow channel, the shape recovery time of two metastatic B16 melanoma variants (B16-F1 and B16-F10) from their compressed state was examined once the cells left the tip of the tapered channel [57]. The B16-F10 cells took longer in recovering their shape than the B16-F1 cells. Considering the fact that B16-F10 cells have higher metastatic potential than B16-F1 cells, the results suggest a role of shape recovery time may be a marker or contributor for the cancer cell’s metastatic potential. The biophysical changes of cells can be used for drug-screening applications.

#### 4.1.2. Simulating Fluid Shear Stress in the Microfluidic-Based On-Chip Models

One of the great advantages of microfluidic devices over the conventional methods is precise mimicking of the in vivo fluidic flow in the channels. Tumor cells are continuously subjected to mechanical forces due to fluid flows caused by their local microenvironmental pressure changes during the metastatic dissemination process. To produce luminal and trans-endothelial flows at physiological magnitudes and to understand the role of shear stresses at different steps of metastasis, extravasation, and interstitial migration, MVN-chips were used [44]. The MVN cancer-chip model consists of three-parallel channels; one or all of which can be used to create the fluid flow that can be controlled by the pressure regulator. Using the MVN-chip, the luminal flow was produced across the MVNs between two side channels while the trans-endothelial flow was produced at the central channel. The luminal flow inside the MVN-chip increased the intravasation of tumor cells, where trans-endothelial flow accelerated the traveling speeds of extravasated tumor cells, which remain in close vicinity with the endothelium.

As a general consideration, cancer cells migrate in the direction of “blood flow”. This was proved by a laminated microfluidic chip built with a vascular cavity with fluid flow [29]. The chip was fabricated from three vertically overlapped PDMS plates and two porous polycarbonate membranes. Both HepG2 liver and MDA-MB-231 breast cancer cells migrated in the direction of “blood flow”. Through a unique multichannel microfluidic model, the impact of hydrodynamic shear stress on EMT and the drug response to A549 lung cancer cells was studied [58]. The model showed flow induced EMT by downregulating E-cadherin expression but increasing N-cadherin and vimentin expressions in lung cancer tumoroids. Distinct reactions to drugs under flow versus static conditions were also observed, indicating the critical role of flow in anti-tumor drug response.

In metastatic breast and prostate cancers, osteocytes and cancer cells communicate through the mechanical stimulation that is induced by fluidic shear stress. Osteocytes generally inhibit metastatic breast and prostate tumor growth and turn metastatic cancer cells towards a mesenchymal phenotype [59]; however, mechanical stimulation by constant high flow rate 1000 µL/h (shear stress ~0.03 Pa) in comparison to standard flow rate 30 µL/h (shear stress ~3 × 10^−5^ Pa) reversed some of these effects. A microfluidic model with oscillatory fluid flow (peak stress of 1 Pa) was developed. A microfluidic-chip model has been developed that consists of a blood vessel with 3D lumen structure through which cancer cells can extravasate and generates oscillatory fluid flow (peak stress of 1 Pa) through which bone cells can produce physiologically relevant mechanical forces to the cancer cells. The model showed enhanced cancer cell migration, proliferation, and invasion in the existence of osteocytes and mechanical stimulation. Paradoxically, it was shown that the shear stress caused by interstitial fluid flow from bone loading exercise differently activated osteocytes in preventing bone metastasis [60].

CTCs, either as single cell or clusters, present with differential metastatic potential. CTC clusters are more invasive than their single counterparts. A novel multichannel microfluidic chip was fabricated to simultaneously reproduce different hemodynamic wall shear stress by increasing vessel branching and the correlation between shear stress and CTC clusters behavior was assessed [61]. Inside the chip, typical wall shear stress values of 2, 5, 20 dyn/cm^2^ was generated that respectively follow the flow rates of capillaries, veins, and arteries. The effects of different wall shear stress on circulating single or clusters of breast cancer cells were examined by injecting the cell suspension into the microfluidic chip. Increasing wall shear stresses was associated with disaggregation of cell clusters; however, with low shear stress, the opposite effect was observed that allowed the isolation of intact CTC clusters. Another study highlights the importance of collective cell migration that is a coordinated, interactive process involving cell–cell and cell–ECM interaction. Using a microfluidic based model, it was detected that randomly distributed pre-existing K14^+^ leader cells migrate from the tumor organoid to “polarize” in response to multiple dynamic changes in the TME, specifically in the presence of chemokine gradients (such as SDF1 gradient) or interstitial fluid flow [54].

The impact of fluid shear stress on CTC’s stiffness, using human glioma cells (U87) as a model cell line, was studied on a straight microfluidic channel that was combined with a live single-cell extractor and atomic force microscope [62]. In general, the cells unexposed to fluid shear stress always exhibited greater nuclear stiffness than cortex stiffness, while after fluid shear stress exposure the cortex hardened, and the nucleus softened. These results indicate that fluid shear stress influences the biomechanics of CTCs. The elucidation of the mechanical responses to fluid shear stress might provide deeper insight into cancer metastasis. CTCs need to pass through a narrow capillary lumen of 4–9 µm in diameter, and then translocate into endothelial cell junctions in 3–10 µm. These studies using microfluidic based models along with in vivo models revealed that the size and deformability of CTC nucleus in response to fluid shear stress, rather than the whole CTC, determine their translocation capability through capillaries and their survival [3].

#### 4.1.3. Simulating Cellular Deformability in the Microfluidic-Based On-Chip Models

Deformability is correlated with the invasion and metastasis of cancer cells. Different casting and fabrication features of microfluidics, such as micropillars, microfilter, or microconstrictions, make it adaptable to study the cellular deformability. A rheological microfluidic system was optimized including different parameters, such as microchannel size, flow rate, and flow viscosity [63]. The cell deformation process was captured by a high-speed camera in this system and the cell deformation index was obtained by an image processing algorithm. The mechanical properties of prostate cancer cell lines (LNCap, PC3, and DU145) were measured: androgen-sensitive prostate cancer cell line LNCaP was less stiff compared to androgen-non-sensitive prostate cancer cell lines (PC3 and DU145). The results indicated that shear stress-induced deformation of prostate cancer cells that can be useful as a potential biomarker for the early diagnosis of androgen-independent prostate cancers. In another study, the fluid flow generated in microfluidic chip induced deformation of metastatic colorectal cancer cells (CRC) that can be used as a physical biomarker for mechanical changes associated with CRC progression [64].

Cellular deformability is correlated with the invasiveness of mesenchymal cancer cells, which was shown by a microfluidic model. A stretchable hydrodynamic microfluidic system was used to make single cells by avoiding cell aggregates and debris [65]. The correlation between deformability and changes in the EMT was investigated using the microfluidic model. RKO, a CRC cell line with the mesenchymal-like feature, had higher deformability than the HCT116 cell line with an epithelial-like phenotype. Moreover, knocking out of TP53 gene from breast epithelial cells caused higher deformability than their isogenic wildtype, suggesting a possible genetic connection to cellular deformability.

Highly metastatic cancer cell lines have higher deformability and plasticity than less metastatic ones under shear flow and mechanical stress. Five different types of microfluidic model with five different geometries were fabricated to test the effect of different mechanical constrictions that is induced by geometry on the flow-induced migration of single CTCs [66]. The “unconfined” and “confined” types in the models had multiple channel heights and constriction widths. Two metastatic breast cancer cells, one mesenchymal-like (MDA-MB-231) and one epithelial-like (SK-BR-3), were streamed into the microfluidic device by controlling the pressure independently. MDA-MB-231 cells showed higher deformability than SK-BR-3 cells in the chips with different geometry.

While the physiological blood pressure is generally much higher than 100 Pa, in vivo, the size and deformability of the nucleus determine whether the CTC can translocate through capillaries. Microfluidic-based model mimicking capillaries and endothelial junctions were used for real-time monitoring of various cell’s translocations through microconstrictions under a wide pressure range [3]. The findings indicated that the cytoplasm decides the total deformation when the transition pressure is lower than ≈100 Pa while the nucleus determines the total deformation when the transition pressure is more than ≈100 Pa. Because the in vitro driving pressure (microfiltration pressure) and in vivo capillary pressure are normally greater than the transition pressure, the deformability and size of the nucleus determine the cell’s travel through the microconstrictions.

A flow-free microfluidic device was developed through a set of circular cross-section pillars for continuous evaluation of cellular deformability and migratory activity [67]. Differing from the popular Boyden transwell chamber with only one pore size, the microfluidic chips can be designed with arrays of circular cross-sectional micropillars with different pore sizes of 7, 5, and 3 µm. Transfecting HeLa cells with a fluorescent Premo FUCCI Cell Cycle Sensor, the cells were seeded into the microfluidic device to study the cell migration in distinct cell cycle phases (G1/S/G2-M). Except for mitosis, there were no differences in migration velocities among cell cycle phases.

#### 4.1.4. Simulating Oxygen Gradients and Hypoxia in the Microfluidic-Based On-Chip Models

Microfluidic-based on-chip cancer models are used to study the effects of tumor oxygen gradients on the movement of cancer cells. A microfluidic chip that can create an oxygen gradient between ambient and hypoxic conditions has been developed maintaining a stable gradient for 24 h [68]. Breast cancer stem cells (CSCs) migrated towards the low oxygen gradient in the microfluidic device. However, a low oxygen gradient alone may not be sufficient to stimulate the metastatic response. Thus, a more complex design was introduced with five channels incorporating two gas channels, two media channels, and a gel channel that can create a durable oxygen tension ramp in the microfluidic device. In this model, cancer cells were directed toward higher oxygen tension and resisted cell death under anticancer drugs [55].

Tumor cells actively migrate toward hypoxic vessels within the TME that act as a guiding cue. A jumbo spheroid-on-a-chip models has been introduced recently that can create a hypoxic 3D TME [69]. Conventionally, hypoxia is artificially induced by a hypoxic chamber, low O_2_ incubator, chemicals, or other chemical inhibitors. The jumbo spheroid-based chip model utilizes tumor spheroids, as large as 750 µm in size, to produce hypoxia and express hypoxic marker protein carbonic anhydrase IX inside the core of the spheroid. The platform can accommodate up to 240 jumbo spheroids. This could be an excellent tool to study the role of tumor hypoxia in metastasis progression and to screen anticancer drugs targeting hypoxia.

## 5. Conclusions, Challenges and Future Perspectives

### 5.1. Conclusions

Tumor metastasis is a complicated process that consists of multiple communicative biochemical and mechanical factors. Invasion/intravasation, circulation, extravasation, colonization, micrometastases, and angiogenesis are widely accepted pathways for the metastasis process. Microfluidic platforms enable study of the metastatic process with unprecedented detail as it offers an opportunity to mimic multiple physiological conditions in a set up through the flexibility of design variations. This review paper elucidates the development of cancer-on-a-chip microfluidic platforms in recent years that are used to study the biochemical and mechanical factors involved in different steps of metastasis.

The microfluidic chips—used for studying invasion/intravasation, extravasation, and angiogenesis—can be broadly categorized based on their chip design and bio-mimicking process. Based on the phase of the media used to study the invasion process, the bio-mimicking microfluidic systems can be classified as: free (fluid-filled) space and 3D hydrogel ECM space. MVN models are promising bio-mimicking systems that utilize the ability of ECs to self-assemble into vascular networks. For invasion/intravasation and extravasation platforms, researchers have mainly focused on developing rectangle lumen vascular platforms. Round luminal vascular channels have also been developed through viscous finger patterning or a microneedle-based removable method. Although the diameter is large, the flow conditions representing physiological flow of healthy vessels of pathological flow of tumoral vessels can be tuned through the design.

The benefits of using microfluidic platforms in studying the cancer metastasis process are apparent from the research articles discussed in this review. Proper use of cancer-on-a-chip model has allowed us to figure out the role of various chemical and physical aspects that directly influence the metastatic steps. However, there are many limiting factors in cancer-on-a-chip models that require further attention.

### 5.2. Challenges and Future Perspectives

Though cancer-on-a-chip platforms have seen considerable development in recapitulating the in vivo microenvironment and systematically simplifying the experimental processes in order to study the process of metastasis, many details are still elusive. The limitations of current microfluidic-based models in elucidating the mechanism of metastasis and their possible solutions are discussed in this section.

1. The mechanism studies carried out so far focus on either exploring the biochemical or mechanical factors. However, both components are responsible for the progression of the metastatic process. Therefore, microfluidic models should be developed in such a way that both of these factors can be studied simultaneously. This could also be done utilizing existing chip models by carrying out studies where biochemical and mechanical cues are introduced in a stepwise manner.
2. Most microfluidic-based cancer-on-a-chip models use buffers to simulate the interstitial flow of the blood stream, which are very different from the physiological condition, such as the rheology of the blood. Whole blood viscosity positively correlates with the cancer stage and metastases [70,71,72,73]. Thus, the blood viscosity should be taken into consideration before flowing into the cancer chips. Recently, another study has shown the impact of red blood cell (RBC) dynamics in the rate of tumor oxygenation, where RBC partitioning at the bifurcations of compressed tumor vessels follow a hematocrit-dependent rather than flow rate-independent manner. Compressed vessels bias red blood cell partitioning at bifurcations in a hematocrit-dependent manner: Implications in tumor blood flow. Thus, the inclusion such versatile blood components would be important to analyze the impact of mechanical factors in tumor progression.
3. Microfluidic platforms where cells invade free (fluid-filled) space are friendly for downstream analysis. However, in most platforms, cells invade into a 3D hydrogel ECM spacer, which preserves cell-ECM interactions, are difficult for downstream analysis. Thus, the technical difficulty of analyzing samples from microfluidic devices to perform imaging, Western blotting, qPCR, single cell RNA sequencing and high-throughput omics should overcome by engineering the cancer-on-chip systems with various degrees of sophistication.
4. PDMS is the most widely used material in the fabrication of microfluidic platform. However, PDMS has several limitations such as: the microstructures are often inaccurate, and it severely absorbs biomolecules. One possible solution could be a microenvironment mimicking material. A bio-based new material—horseradish peroxidase crosslinked silk fibroin has been developed [74]. This new silk fibroin hydrogel has advantages at tuning mechanical properties, such as stiffness. Therefore, utilizing different biocompatible materials and crosslinking agents could provide a solution to the issue of using PDMS.
5. Vessel angiogenesis is the marker for forming secondary tumors. The currently used TME mimicking models, other than observed vasculogenic mimicry, have been utilized for studying sprouting angiogenesis [36,37,41,42]. To the best of the authors’ knowledge, no cancer-on-a-chip has been reported to study the other mechanisms: vessel cooption, vascular mimicry, and intussusceptive angiogenesis as shown in Figure 2 [75]. To study the other angiogenesis processes, microfluidic chips will need to be designed in specific ways.
6. Preparation and maintenance of 3D culture microfluidic chips are still time-consuming processes. Generally, several hours to days are required for fabrication, modeling, culture, and obtaining the results. The more factors that are introduced, the more precise the control that is needed. Also, to mimic in vivo TME, a lot of time and effort is needed. Implementing digital control systems for this kind of complex processes could ensure precise control and minimize human effort.
7. Most studies utilize existing chip models. In some respects, this limits the possibilities that the newer design could offer. Researchers should focus on designing chips that can be used to address multiple factors of the metastasis process.
8. Due to the inclusion of artificial ECM components or lack of primary features, present platforms are restrained from working with primary cells or tissues. Some recent studies have involved the patient-derived samples replicating personal physiologically relevant TME [24]. In the future, more attention should be paid towards studying primary cells including cancer cells, CAFs, and models involving immune cells for tailored TME studies.

## Figures and Tables

**Table 1:** Cancer-on-a-chip microfluidic platforms.

Models	Cues	Cancer Type	Microfluidic Features	General Outcomes	Ref.
Invasion/Intravasation	Inflammatory Cells	Breast cancer	A juxtaposed dual-layer cell-loaded hydrogels biomimetic microfluidic system	Tumor-associated macrophages (TAM) phenotyping was maintained by breast cancer cells while breast cancer cells promoted the differentiation of U937 cells into TAM.	[19]
		Lung cancer	A microfluidic-based co-culture device with two polydimethylsiloxane (PDMS) layers sandwiching a transwell membrane segmented into two chambers	M2 macrophages upregulated CRYAB expression and activated the ERK1/2/Fra-1/slug signaling pathway to promote epithelial-to-mesenchymal transition (EMT) and malignancy of lung cancer cells.	[20]
		Ovarian Cancer	Micro-culture device with a PDMS ring	Activated macrophages secreted MIP-1β that activated CCR5/PI3K signaling in mesothelial cells and induced P-selectin expression on the cell surface to promote ovarian cancer adhesion.	[21]
		Breast cancer	A central microchamber where cancer cells with/without NK cells can be grown that is connected to a vessel at one corner of the chip	The process of natural killer (NK) cell exhaustion was shown in response to tumor microenvironmental stresses.	[22]
		Breast Cancer	A three-channel microfluidic system mimicking the lymph vessel-tissue-blood vessel structure	Vascular endothelial growth factor (VEGF) secreted by HLECs upon IL-6 stimulation caused the HUVECs to grow inside the cancer cell clusters that are located near the lymphatic channel.	[23]
	CAFs	Breast Cancer	3D co-culture organotypic invasion model for crosstalk of fibroblasts and cancer cells	Cancer-Associated Fibroblasts (CAFs) enhanced invasion by inducing gene expression of glycoprotein non-metastatic B.	[24]
		Breast cancer	LumeNEXT microfluidic model	Both metalloproteinases (MMP) and fibronectin were essential for the invasion of MDA-MB-231 cells.	[25]
		Breast cancer	A 3D-printed brick like cell patterning microfluidic platform	The tumor cells and fibroblasts crucially impacted each other.	[26]
		Colon and breast cancer	A microwell array-based microfluidic platform	Tumor spheroids could envelop fibroblast spheroids completely that helped the colon cancer cells to invade at short time.	[27]
		Melanoma, squamous and breast cancer	3D five-channel model that allows co-culturing CAFs and cancer cells with real-time monitoring of invasion process	The invasive area of cancer cells into the ECM in the presence of exosomes-induced CAFs was higher than exosome non-treated CAFs.	[28]
	Endothelium based models	Breast and liver cancer	A vascular cavity with fluid flow in the laminated microfluidic chip	MDA-MB-231 breast cancer cells invaded paracellularly by disrupting the intercellular endothelial junction, whereas HepG2 liver cancer cells invaded through the transcellular process.	[29]
		Breast cancer	Invasive ductal carcinoma-on-chip by viscous fingering	MCF-7 cell line was non-invasive and non-tumorigenic unless supplemented with estrogen whereas TNB subtypes invaded into the surrounding matrix.	[30]
		Ovarian cancer	Ovarian TME organ-on-chip platform	Platelets promote ovarian cancer invasion by interactions between glycoprotein Ⅵ and tumor galectin-3 under shear.	[31]
Extravasation		Breast cancer and fibrosarcoma		Cdk5 affects vascular adhesion, structure of Tln1 and FAK supports invadopodia formation while FAKS732 phosphorylation participates in trans-endothelial migration.	[32]
		Breast Cancer		The glycocalyx defects during extravasation from perfusable endothelial lumens was visualized.	[33]
		Breast Cancer	Microfluidic chip with three to five microchannels that can form microvascular networks	Trans-endothelial migration and invasion of cancer cells occur through binding CD44 to the sub-endothelial ECM components.	[34]
		Melanoma		Lipopolysaccharide (LPS)-stimulated neutrophils aggregate under flow, and arrest by mechanical trapping and interactions with endothelial ICAM-1 into the vascularized channels.	[4]
		Breast Cancer		Induced by hypoxia, both HIF-α protein level and rate of cancer cells extravasation increased.	[35]
Angiogenesis			Three-lane microfluidic titer plates	A combination of VEGF-165, PMA, and S1P was the foremost optimum cocktail to trigger vigorous and directional angiogenesis.	[36]
		Cervix cancer	A tumor spheroid-based microfluidic device	At an extremely early stage of cancer, endotheliocytes and fibroblasts accelerated the proliferation and migration of HeLa cells in chips while vasculogenic mimicry was observed.	[37]
			MVN-chip	Effect of interstitial flow in vascular sprouting cannot be substituted by increasing vascular endothelial growth factor.	[38]
		Colorectal and gastric cancer	3D microfluidic bone model with hydroxyapatite acid stimulating mechanical properties of bone	During angiogenesis in the HA/fibrin composite, the number of blood vessel sprouts decreased as the HA concentration increased.	[39]
			Multi-tissue chamber model with one central chamber adjacent to other chambers and two lateral media lines	Increased levels of CAF mechanical activity contributed to increased angiogenesis.	[40]
		Breast cancer	Microfluidic organ-on-a-chip models of solid tumors	Culturing MDA-MB-231/HUVECs in a HLFs-laden, fibrin-based ECM promoted angiogenesis and tumor cell migration.	[41]
		Inflammatory breast cancer (IBC)	3D in vitro vascularized microfluidic based inflammatory breast cancer model	IBC platforms increased collagen ECM porosity and expressed higher levels of VEGF than non-IBC and control.	[42]

**Table 2:** Microfluidic modeling: A focus on mechanical factors in cancer cell migration.

Mechanical Factors	Cancer Type	Microfluidic Features	General Outcomes	Ref.
Stiffness	Breast cancer	Microfluidic devices contained three independently addressable parallel channels	Cancer cells increase their extravasation capability in regard to the substrate stiffening which could be connected with the expression level of metalloproteinase-9 (MMP-9)	[56]
	Melanoma	A linearly tapered microflow channel	Test cancer cells’ shape recovery time after compressed compared to relative invasive ability.	[57]
Shear stress	Breast cancer	microvascular network (MVN)-chip	The luminal flow increased the intravasation, where trans-endothelial flow increased migratory speeds of extravasation.	[44]
	Breast and liver cancer	A vascular cavity with fluid flow in the laminated microfluidic chip	Cancer cells migrate in the direction of “blood flow”.	[29]
	Lung cancer	A multichannel microfluidic model	Flow induced EMT by decreasing E-cadherin expression but increasing N-cadherin and vimentin expressions.	[58]
	Breast and prostate cancer	Microfluidic organ-chip using two overlapped microchannels separated by a membrane	Mechanical stimulation by constant high flow rate in comparison to standard flow rate reversed the inhibition metastatic effects.	[59]
	Breast cancer	A novel microfluidic cancer extravasation tissue platform	The shear stress caused by interstitial fluid flow from bone loading exercise prevents bone metastasis.	[60]
	Breast cancer	A novel multichannel microfluidic device simultaneously reproduces different hemodynamic wall shear stress	Increasing shear stress was associated with disaggregation of cell clusters while low shear stress was associated with the opposite effect.	[61]
	Breast cancer	Three parallel tissue chambers surrounded by two parallel microfluidic lines	Pre-existing K14+ leader cells travel through the organoid to “polarize” to the front rim in regard to SDF1 gradient, and interstitial flow.	[54]
	Brain cancer	A straight microfluidic channel combined with a live single-cell extraction and atomic force microscopy	The cells unexposed to fluid shear stress exhibited greater nuclear stiffness than cortex stiffness, while after fluid shear stress exposure the cortex hardened, and nucleus softened.	[62]
Cellular deformability	Prostate and colorectal cancer	Microfluidic hydrodynamic stretching with high-speed capturing camera	Shear stress-induced deformation as potential biomarkers of early detection or metastatic progression.	[63,64]
	Colorectal and breast cancer	A stretchable hydrodynamic microfluidic system with microfilter	The mesenchymal-like cells had higher deformability than the epithelial-like cells.	[65]
	Breast cancer	Five types of geometric microfluidic models	Highly metastatic cancer cell lines have higher plasticity and deformability under shear flow and mechanical stress.	[66]
	Breast cancer	Microconstriction array mimicking capillaries and endothelial junctions in a microfluidic device with real-time monitoring	The deformability and size of the nucleus determine the cell’s translocation through the microconstrictions.	[3]
	Cervix cancer	Microfluidic arrays of circular cross-section micropillars with decreasing spacing	Except for mitosis, there were no difference in migration velocities among cell cycle phases.	[67]
Oxygen gradient and hypoxia	Breast cancer	The microfluidic chip with simulated oxygen gradient.	No changes in the migration pattern of breast cancer stem cells (CSCs) were observed from the average cancer cell population.	[68]
	Breast cancer	A microfluidic device with five channels including two gas channels, two media channels, and a gel channel	Cancer cells direct toward higher oxygen tension and resist cell death against anticancer drug.	[55]
	Sarcoma	Hypoxic jumbo spheroids on-a-chip	Validate the establishment of the device and jumbo spheroids.	[69]
